# Analyzing the Driving Forces of Vegetation Change in the Yellow River Basin: Comprehensive Assessment of Natural Social and Economic Factors

**DOI:** 10.3390/plants15040628

**Published:** 2026-02-16

**Authors:** Fei Gao, Ying Yang, Weijie Yuan, Xiuxiu Deng, Lina Wang, Shuai Zhang

**Affiliations:** 1 Experimental Center of Forestry in North China, Chinese Academy of Forestry, Beijing 102300, China; gaof.20b@igsnrr.ac.cn (F.G.); yangying@caf.ac.cn (Y.Y.); yuanwj@caf.ac.cn (W.Y.); dengxiuxiu@caf.ac.cn (X.D.); wanglina931@caf.ac.cn (L.W.); 2 National Permanent Scientific Research Base for Warm Temperate Zone Forestry of Jiulong Mountain in Beijing, Beijing 102300, China; 3 Key Laboratory of Ecosystem Network Observation and Modeling, Institute of Geographic Sciences and Natural Resources Research, Chinese Academy of Sciences, Beijing 100101, China

**Keywords:** leaf area index, driving mechanisms, Geographically and Temporally Weighted Regression, ecological restoration

## Abstract

Leaf area index (LAI) is a key vegetation structural parameter widely used to quantify vegetation dynamics. A thorough understanding of its spatiotemporal characteristics and driving mechanisms is essential for sustainable ecosystem management. This study combines LAI and climate remote sensing data with socioeconomic statistics, and uses the Geographically and Temporally Weighted Regression (GTWR) model and geographic detector to identify the key drivers of LAI changes and their spatial differentiation characteristics. The results indicate a significant upward trend in the LAI across the basin, with a markedly higher growth rate after 2000 (0.0123/year) compared to the period before 2000 (0.0028/year). Spatially, before 2000, 57% of the regions showed an increasing trend in LAI, while after 2000, 69% of the regions exhibited an increasing trend in LAI. In terms of temporal LAI dynamics, in the eastern region, the positive promotion effect of the Grain for Green policy (GRGR) was the most significant factor affecting LAI changes. In the central region, the employed population ratio (EPR) emerged as the dominant factor driving LAI increase. In the western region, the negative effect of the urban–rural income ratio (IR) served as the primary driver of LAI change. Regarding the spatial distribution of LAI, both natural and policy factors were statistically significant. Mean precipitation (MP) and mean slope (MS) exerted the most pronounced influences, followed by mean temperature (MT) and GRGR, whereas mean elevation (MD) showed the weakest effect. Notably, socioeconomic factors did not demonstrate statistically significant impacts on the spatial distribution of LAI. This study provides a theoretical foundation for understanding the driving mechanisms of LAI dynamics in the Yellow River Basin and offers scientific support for ecological restoration and sustainable management in the region.

## 1. Introduction

Vegetation, a core component of terrestrial ecosystems, plays a vital role in energy exchange, carbon cycling, and water circulation across the Earth’s surface [1,2], while also providing essential material foundations for socioeconomic development [3,4]. Vegetation growth is influenced by both natural factors and human activities through highly complex mechanisms [5,6]. In recent years, intensifying global environmental change [7,8] and the large-scale implementation of ecological projects [9,10] have driven marked changes in vegetation dynamics [11]. Against this backdrop, gaining an in-depth understanding of the characteristics and driving mechanisms of vegetation dynamics is crucial for the sustainable management of ecosystems.

Current research on the drivers of vegetation change primarily focuses on two aspects: natural factors and human activities. Among natural factors, precipitation and temperature are the most widely studied [12,13]. Previous studies have shown that rising temperatures significantly promote greening trends in high-latitude and high-altitude regions by enhancing photosynthesis and extending the growing season [14,15]. In contrast, in arid regions, increased temperatures can suppress vegetation growth by elevating evapotranspiration [16]. Precipitation also exhibits a dual influence on vegetation dynamics: in water-limited regions, increased precipitation generally promotes growth [17], whereas in some areas, exceptionally wet years may lead to waterlogging or other stressors that inhibit vegetation development [18,19]. Regarding the impact of human activities, most existing studies utilize residual-based methods to attribute vegetation changes to human influence as an aggregated factor. For example, Chang et al. [9] analyzed the vegetation greening trend in China from 2001 to 2020 and used a residual approach to quantify the contributions of climate and human activities at 21.89% and 78.11%, respectively. Peng et al. [20] applied residual threshold analysis between leaf area index and climate variables to identify regions where vegetation changes in China’s karst areas were dominated by human activities versus climate change. Zhang and Ye. [21] also used an improved residual method to separate the effects of climate change and human activities on vegetation across mainland China. Some studies equate human activities primarily with ecological engineering projects; for instance, Song et al. [22] analyzed the effects of temperature, precipitation, and ecological restoration projects on vegetation dynamics in China, concluding that human activities were the dominant driver behind the observed vegetation greening. However, human activities constitute a complex process that should encompass multidimensional factors representing socioeconomic conditions. Currently, a limited number of studies have attempted to broaden the characterization indicators of human activities [23]: Pang et al. [24] considered factors such as population density, economic density, and nighttime lights in studying vegetation dynamics in the Taihang–Yanshan region, the study by Zhou et al. [25] revealed that variables such as car ownership rates, conservation awareness, and agricultural practices exerted significant influences on vegetation restoration. Nevertheless, the impact of socioeconomic factors on vegetation restoration has not yet been comprehensively addressed, research in this area remains in its early stages and requires further in-depth exploration.

The Yellow River Basin serves as a critical ecological barrier for maintaining ecological balance in northern China [26]. This region features complex climatic and topographic conditions and is environmentally fragile [27]. Due to overgrazing in the 1980s and 1990s, grassland degradation was widely reported in the basin [28]. To address ecosystem degradation, the government implemented the Grain for Green Program starting in 1999, converting large areas of rain-fed cropland to grassland and forest [29,30]. In recent years, widespread vegetation greening has been observed across the Yellow River Basin. For example, Xiao et al. [31] reported that the Grain for Green Program increased forest cover in low-elevation areas of the basin by 41%, and Tian et al. [32] showed that since the beginning of the 21st century, over 94% of the basin has experienced vegetation greening, with more than 71% exhibiting a significant greening trend. These studies attributed the vegetation greening primarily to the Grain for Green Program. However, recent research suggests that the positive effects of the Grain for Green Program on vegetation may be short-term and limited [6,25], while socioeconomic factors such as economic development and population dynamics may have more fundamental impacts on vegetation greening [33]. Moreover, these factors evolve with regional environmental policies and economic growth. Only a few studies have examined the role of socioeconomic drivers in vegetation recovery in the Yellow River Basin. For instance, Li et al. [34] found that in the Loess Plateau, socioeconomic factors had a more pronounced influence on vegetation restoration in 47.02% of the area. Naeem et al. [29] investigated both individual and combined effects of climatic and socioeconomic factors on vegetation trends across 294 counties in the Loess Plateau. Although these studies have significantly enriched our understanding of the impact of human activities on vegetation restoration, research in this area remains limited and has not yet reached a consensus. Against this backdrop, our study analyzes vegetation change drivers across four dimensions—natural, social, economic, and policy, aiming to clarify the effects of both natural and human factors on vegetation restoration in the Yellow River Basin.

In summary, while existing studies have predominantly focused on the influence of climatic factors and ecological restoration projects on vegetation dynamics, the contribution of socioeconomic factors remains insufficiently clarified [35], thereby limiting a systematic understanding of the mechanisms driving vegetation changes. The LAI is defined as the sum of the one-sided area of all leaves per unit land area [35], and is often used to describe the structure and function of the vegetation canopy. It has been widely used in quantitative studies of vegetation changes [36]. This study focuses on the middle and upper reaches of the Yellow River Basin, exploring the drivers of LAI variation from 1980 to 2019 using the GTWR and Geographical Detector. The research aims to provide a scientific basis for ecological management in the region. The specific objectives are: (1) to analyze the spatiotemporal variations and spatial heterogeneity of LAI, climatic, social, economic, and policy factors in the Yellow River Basin between 1980 and 2019; (2) to elucidate the drivers of temporal changes in LAI from climatic, social, economic, and policy perspectives; and (3) to assess the influence of these four categories of factors on the spatial distribution of LAI. The findings are expected to offer critical theoretical insights and practical guidance for formulating ecological conservation policies and adaptive management strategies in the Yellow River Basin.

## 2. Materials and Methods

### 2.1. Study Area

**Figure 1:**
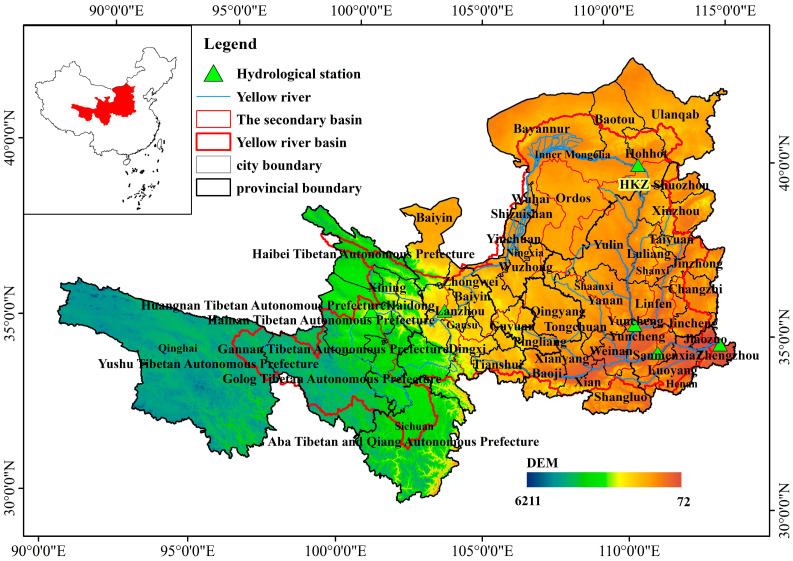
The location of study area. LYX: Longyangxia; LZ: Lanzhou; HKZ: Hekouzhen; SMX: San-menxia; HYK: Huayuankou).

The middle and upper reaches of the Yellow River flow through eight provinces (autonomous regions) in northern China, including Qinghai, Sichuan, Gansu, Ningxia, Inner Mongolia, Shaanxi, Shanxi, and Henan, with a total basin area of 730,000 km^2^ (Figure 1). The upper reach extends from the river source to Toudaoguai hydrological station, spanning a length of 3471 km and covering a drainage area of 386,000 km^2^. The middle reach runs from Toudaoguai hydrological station to Huayuankou hydrological station, with a length of 1206 km and a basin area of 344,000 km^2^ [37]. This study focuses on the administrative boundaries of 50 cities within the middle and upper reaches of the Yellow River as the research scope. The western part of the basin is characterized by high plateaus, while the eastern region consists largely of loess hilly terrain with severe soil erosion. The basin experiences a predominantly arid to semi-arid continental monsoon climate, with an average annual precipitation of approximately 500 mm and mean annual temperatures ranging from −20 °C to 16 °C. The topography slopes from higher elevations in the west to lower areas in the east, and the primary land cover types include grassland, deciduous broadleaf forest, and cropland [38].

### 2.2. Data

The LAI data used in this study were obtained from the Global Land Surface Satellite [39] product service platform “http://glass-product.bnu.edu.cn/” (accessed on 1 September 2025). Precipitation and temperature datasets were sourced from the National Earth System Science Data Center “http://www.geodata.cn/” (accessed on 21 July 2025). Land use data and digital elevation model (DEM) data were acquired from the Resource and Environment Science Data Center “https://www.resdc.cn” (accessed on 12 August 2025). Socioeconomic data were derived from statistical yearbooks of prefecture-level cities.

Socioeconomic factors were compiled at the prefecture-city level. A total of 20 indicators were collected, including total population, urban population, rural population, gross domestic product, gross output value of the primary, secondary, and tertiary industries, employed population in the primary, secondary, and tertiary industries, grain yield, sown area of grain crops, urban disposable income per capita, rural net income per capita, fiscal revenue and expenditure, total retail sales of consumer goods, and fixed-asset investment. To minimize the impact of area and population size on regional comparisons, all indicators were normalized on a per capita and per unit area basis [40]. Descriptions of each variable are provided in Table 1.

Policy factors were represented by the ratio of cumulative area under the Grain for Green Program to total cropland area, derived from land use data. First, areas consistently classified as cropland from 1980 to 2000 were identified as permanent cropland. Then, areas converted to forest, shrubland, or grassland during 2001–2020 were extracted as regions affected by the Grain for Green Program implementation [41].

### 2.3. Methods

#### 2.3.1. Geographically and Temporally Weighted Regression

Geographically Weighted Regression Model (GWR) is a local regression technique that incorporates geographical location information [42], It addresses spatial non-stationarity by decomposing global statistics into local estimates [43], enabling the calculation of relationships between independent variables and the predicted variable at each spatial location. GTWR is a regression model that incorporates the time dimension on the basis of GWR [44], capable of simultaneously depicting the heterogeneity of the influence of explanatory variables due to both spatial location and temporal change. The equation is as follows:

(1) $${y_{i}=\beta }_{0}\left(u_{i},v_{i},t_{i}\right)+\sum_{k=1}^{p}\beta_{k}\left(u_{i},v_{i},t_{i}\right)x_{ik}+\varepsilon_{i}$$

In the formula: $\left(u_{i},v_{i},t_{i}\right)$ represents the spatiotemporal coordinates at location i; $u_{i}$, $v_{i}$, respectively represent the longitude and latitude coordinates at spatial location i, $t_{i}$ represents time, $y_{i}$, $x_{ik}$ and $\varepsilon_{i}$ represent the dependent variable, the k independent variable, and the error term, respectively; $\beta_{0}\left(u_{i},v_{i},t_{i}\right)$ is the intercept; $\beta_{k}\left(u_{i},v_{i},t_{i}\right)$ refers to the regression coefficient of the k independent variable at spatial location i.

#### 2.3.2. Geographical Detector

Spatial heterogeneity is a fundamental characteristic of geographical phenomena. The Geographical Detector model is a statistical method designed to identify spatial stratified heterogeneity and reveal the underlying driving forces [45]. In this study, we utilized the factor detector module in the Geographical Detector to assess the individual influence of each factor on the spatial distribution of LAI.

The calculation is based on the following model:

(2) $$q=1-\frac{\sum_{h=1}^{L}N_{h}\sigma_{h}^{2}}{N\sigma^{2}}=1-\frac{SSW}{SST}$$

In the formula: 0 < q < 1. A larger value of q indicates a stronger influence of the factor; h represents the stratification of each variable; $N_{h}$ denotes the number of units in stratum h, N is the total number of units in the entire region; $\sigma_{h}^{2}$ represents the variance of stratum h, $\sigma^{2}$ is the variance of the entire region; SSW denotes the within-stratum sum of variances; and SST represents the total variance across the entire region.

## 3. Results

### 3.1. Spatiotemporal Variations in Natural and Socioeconomic Factors

#### 3.1.1. Spatiotemporal Variations in Natural Factors

Hydrothermal conditions and topographic features are key environmental factors influencing LAI growth and distribution. This study selected precipitation and temperature to represent climate conditions, and elevation and slope to represent topographic conditions, as the natural environmental factors affecting LAI change. Given that elevation and slope do not change significantly over short time periods, this study considered them only in the analysis of spatial distribution patterns.

**Figure 2:**
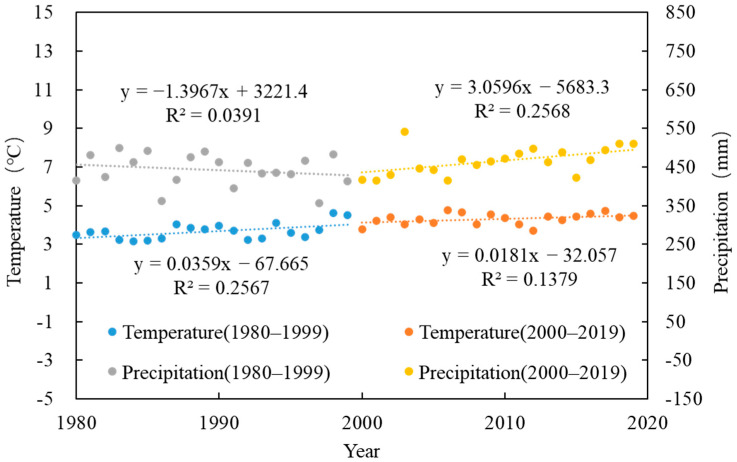
Temporal variations in temperature and precipitation.

The temporal variations in temperature and precipitation in the Yellow River Basin are shown in Figure 2. Temperature exhibited an overall increasing trend, with an average rate of increase of 0.036 °C/year during 1980–2000 and 0.018 °C/year during 2000–2019, indicating that the warming rate before 2000 was twice that after 2000. Precipitation showed an initial decreasing trend followed by an increase from 1980 to 2020, with a decline rate of 1.4 mm/year before 2000 and an increase rate of 3.1 mm/year after 2000.

**Figure 3:**
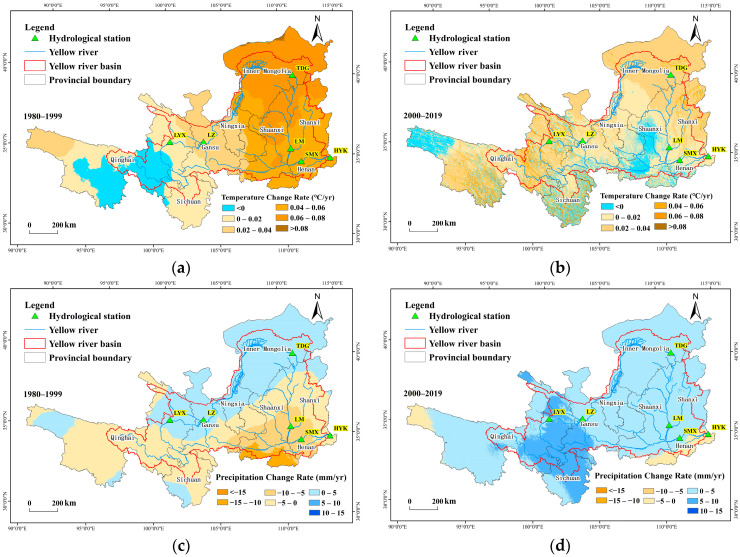
Spatial distribution of changes in temperature and precipitation. (**a**) spatial distribution of temperature changes before 2000; (**b**) after 2000; (**c**) spatial distribution of precipitation changes before 2000; (**d**) after 2000.

The spatial patterns of temperature and precipitation changes from 1980 to 2019 are illustrated in Figure 3. During 1980–1999, temperature exhibited an increasing trend across 88% of the Yellow River Basin, with the rate of increase decreasing from northeast to southwest. A significant warming trend was observed in 52% of the area, primarily located in Inner Mongolia and Shanxi Province, while 12% of the region showed a non-significant decreasing trend. In the period 2000–2019, the temperature increased by 85% of the basin, but only 19% of the area showed a statistically significant increase. Regarding precipitation, from 1980 to 1999, the rate of precipitation change increased from southeast to northwest. Increasing trends were observed in Inner Mongolia, Ningxia, and parts of northern Gansu and Qinghai, whereas decreasing trends occurred in southeastern regions such as Shanxi, Shaanxi, and Henan. Areas with decreasing precipitation accounted for 42% of the total basin area, while those with increasing precipitation accounted for 58%. During 2000–2019, precipitation increased in 95% of the Yellow River Basin, with 21% of the area showing a significant increase, mainly concentrated in the border region of Qinghai, Gansu, and Sichuan provinces, where the increase rate reached 5–10 mm/year. The remaining areas experienced an increase rate of 0–5 mm/year.

#### 3.1.2. Spatiotemporal Changes in the Socioeconomic System

**Figure 4:**
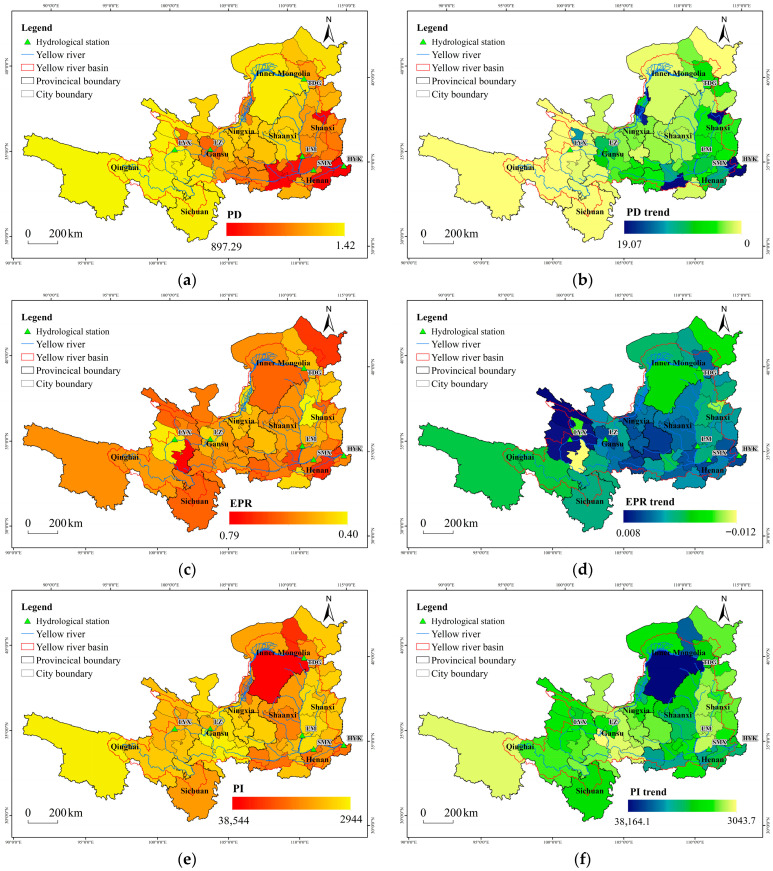

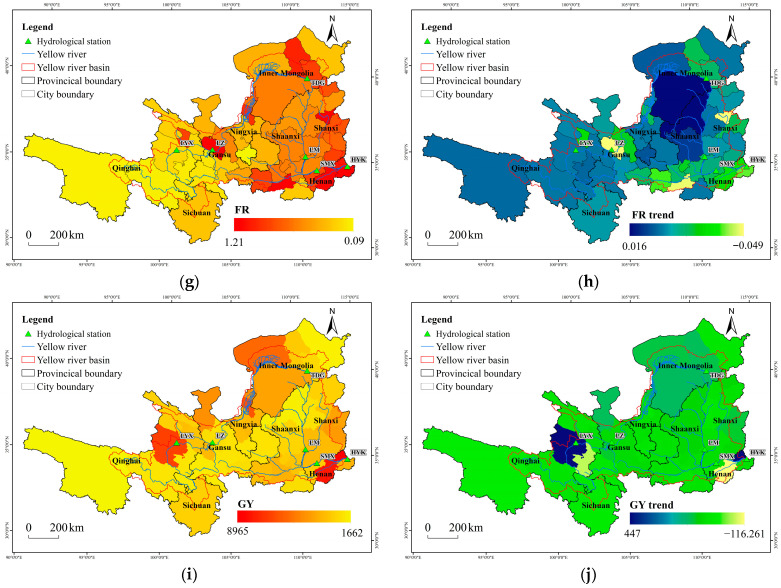
Spatial distribution and changes in social factors. (**a**) spatial distribution of PD; (**b**) spatial distribution of PD changes; (**c**) spatial distribution of EPR; (**d**) spatial distribution of EPR changes; (**e**) spatial distribution of PI; (**f**) spatial distribution of PI changes; (**g**) spatial distribution of FR; (**h**) spatial distribution of FR changes; (**i**) spatial distribution of GY; (**j**) spatial distribution of GY changes.

Spatial distribution and temporal changes of social factors in the Yellow River Basin are shown in Figure 4. PD in the basin ranged from 1.42 to 897 persons/km^2^. The PD was primarily distributed along the river in the middle reaches (Figure 4a,b), with the Longmen–Huayuankou segment having the highest density. The PD growth rate varied between 0 and 19.07 persons/year; faster growth occurred in regions with already high density. Cities such as Xi’an, Taiyuan, Yinchuan, and Zhengzhou showed the most rapid population increases, indicating a trend of population concentration in more developed urban centers. The EPR ranged from 40% to 79%, with no clear spatial distribution pattern (Figure 4c,d). Its temporal change fluctuated between −1.2% and 0.8%. PI fell between 2944 and 38,544 CNY/person. Shaanxi, Henan, and Inner Mongolia had higher values (Figure 4e,f) and also exhibited the fastest growth in this indicator. FR across the basin ranged from 9% to 121% (Figure 4g,h). Eastern and southern regions showed higher ratios but slower growth, whereas northwestern regions experienced more rapid increases in this ratio. GY varied from 1662 to 8965 kg/ha. The highest GY was observed in the Ningxia irrigated area, the Hetao irrigated area, and the regions of Luoyang, Jiaozuo, and Jiyuan (Figure 4i,j), followed by the Guanzhong and Fenhe irrigation districts, indicating that humid climate and irrigation conditions support higher agricultural productivity. The rate of increase in GY showed relatively small variation across the basin.

**Figure 5:**
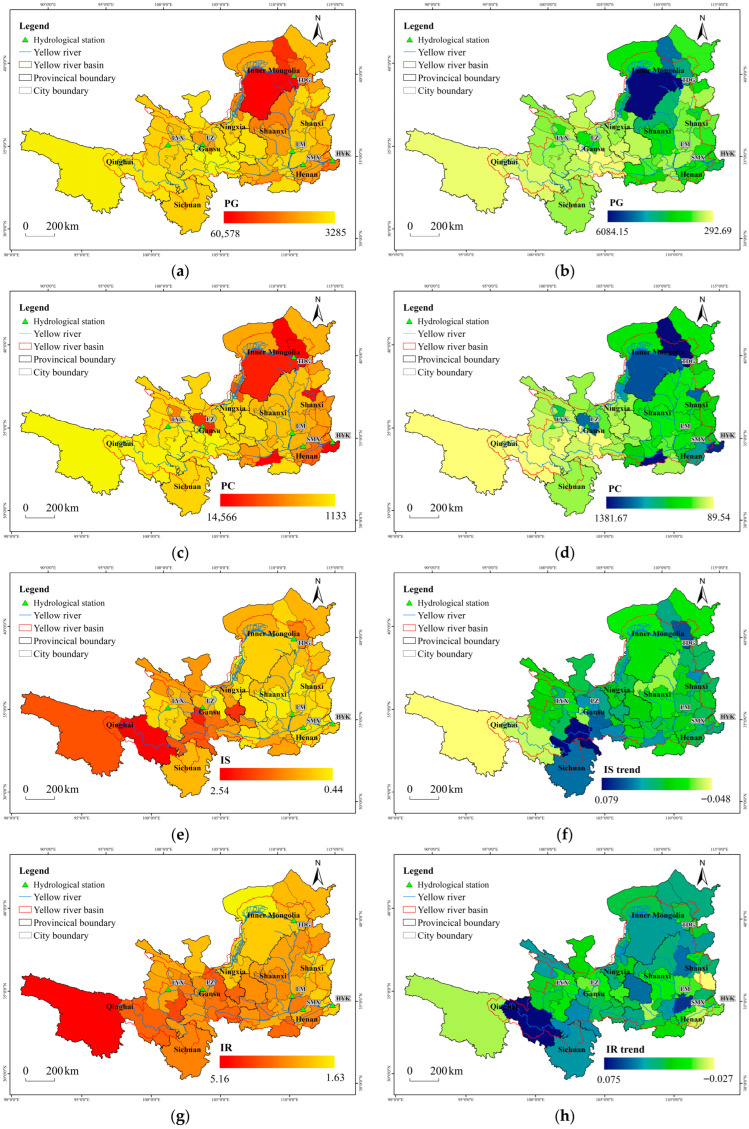

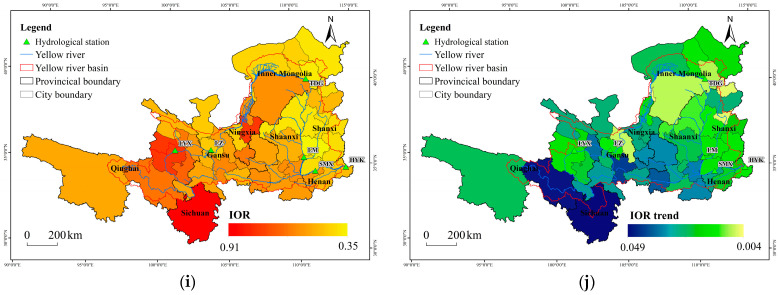
Spatial distribution and changes in economic factors. (**a**) spatial distribution of PG; (**b**) spatial distribution of PG changes; (**c**) spatial distribution of PC; (**d**) spatial distribution of PC changes; (**e**) spatial distribution of IS; (**f**) spatial distribution of IS changes; (**g**) spatial distribution of IR; (**h**) spatial distribution of IR changes; (**i**) spatial distribution of IOR; (**j**) spatial distribution of IOR changes.

Spatial distribution and temporal changes of social factors in the Yellow River Basin are shown in Figure 5. PG among cities in the basin ranged from 3285 to 60,578 CNY/person, with Shaanxi and Henan provinces significantly higher than others (Figure 5a,b). Provincial capitals such as Xi’an, Zhengzhou, Taiyuan, Xining, and Laning had notably higher PG, and these more developed cities also grew faster, suggesting an agglomeration effect in economic development. PC ranged from 1133 to 14,566 CNY/person, with higher values in the middle reaches compared to the upper reaches (Figure 5c,d). The growth rate of this indicator showed a similar spatial pattern. IS is shown in Figure 5e,f. Western regions exhibited more advanced industrial structures and a faster upgrading rate. The IOR ranged between 0.35 and 0.91, indicating higher economic efficiency in the western regions (Figure 5g,h), which also experienced more rapid growth in efficiency. IR varied from 1.63 to 5.16. The region between Lanzhou and Toudaoguai showed relatively lower value (Figure 5i,j), which may be attributed to the prevalence of irrigated agriculture in this area.

#### 3.1.3. Spatiotemporal Changes in GRGR

**Figure 6:**
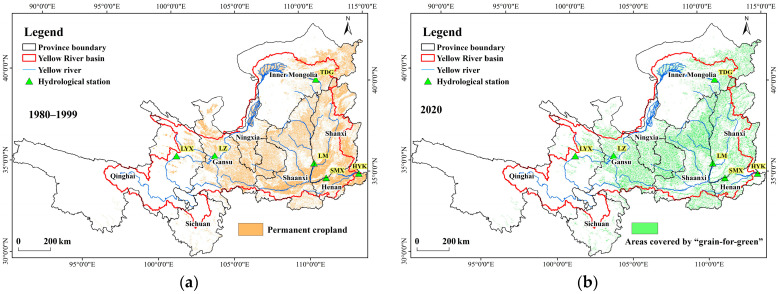
Spatial distribution of permanent cropland and areas under the Grain for Green Program. (**a**) spatial distribution of permanent cropland; (**b**) spatial distribution of areas under the Grain for Green Program.

Since 1999, large-scale Grain for Green Program projects have been implemented in the Yellow River Basin, resulting in the conversion of extensive cropland to forest and grassland, and causing significant changes in land use types. The distribution of permanent basic cropland is shown in Figure 6a, primarily located in Gansu, Shanxi, Shaanxi, Henan, and Ningxia provinces. After 2000, substantial cropland conversion took place. By 2020, a total of 92,900 km^2^ of cropland in the middle and upper reaches of the Yellow River had been transformed into forest, shrubland, or grassland. The spatial distribution of cumulative area under the Grain for Green Program projects is illustrated in Figure 6b.

**Figure 7:**
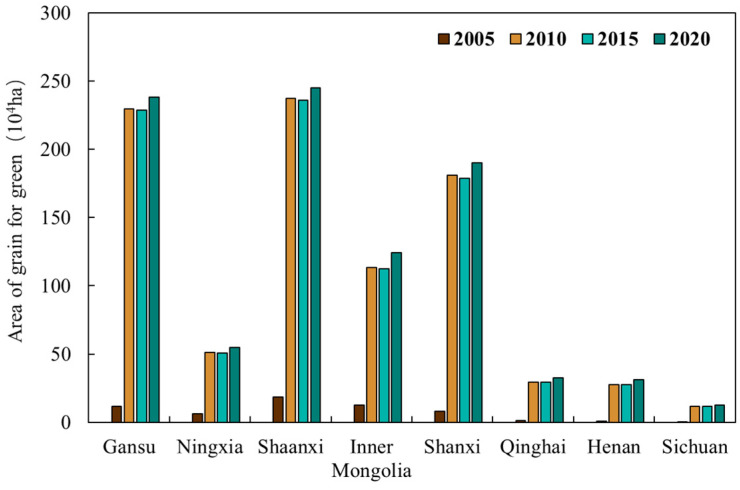
Cumulative area of the Grain for Green Program by province.

Figure 7 shows the cumulative area under the Grain for Green Program by province in the Yellow River Basin. Shaanxi and Gansu provinces had the largest implemented area under the Grain for Green Program, each exceeding 2 million hectares. They were followed by Shanxi, with over 1.5 million hectares, and Inner Mongolia, with more than 1 million hectares. The implemented areas in Ningxia, Qinghai, Henan, and Sichuan were each below 500,000 hectares. In terms of temporal changes, all provinces exhibited a highly consistent implementation pattern. Before 2005, the area enrolled in the Grain for Green Program was relatively small, accounting for less than 10% of the total ultimately converted. From 2005 to 2010, the area under the program expanded rapidly, reaching over 90% of the total implemented area. Implementation was largely suspended between 2010 and 2015, during which the cumulative area remained nearly unchanged. A slight increase resumed from 2015 to 2020. The large-scale implementation of the Grain for Green Program was primarily completed during the period 2005–2010.

**Figure 8:**
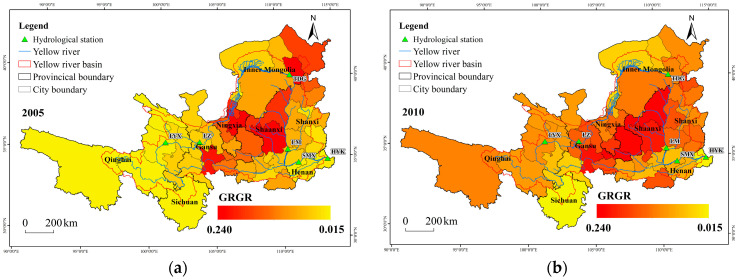

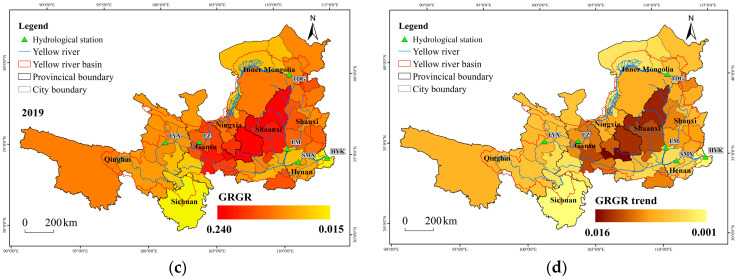
Spatial distribution and changes in GRGR. (**a**) spatial distribution of GRGR in 2005; (**b**) in 2010; (**c**) in 2019; (**d**) spatial distribution of GRGR changes.

This study used the ratio of cumulative area under the Grain for Green Program to total cropland area to measure the implementation intensity of the program. Its temporal and spatial variations are shown in Figure 8. The ratio of Grain for Green Program area at the city level in the Yellow River Basin ranged from 1.5% to 24%. Regions with higher implementation intensity were mainly located in the Loess Plateau within the middle reaches of the Yellow River, particularly in Shaanxi and Gansu provinces, followed by Shanxi. From 2000 to 2019, the growth rate of GRGR across the basin varied between 0.1% and 1.6%, with Shaanxi exhibiting the fastest increase.

### 3.2. Spatiotemporal Variations in LAI

**Figure 9:**
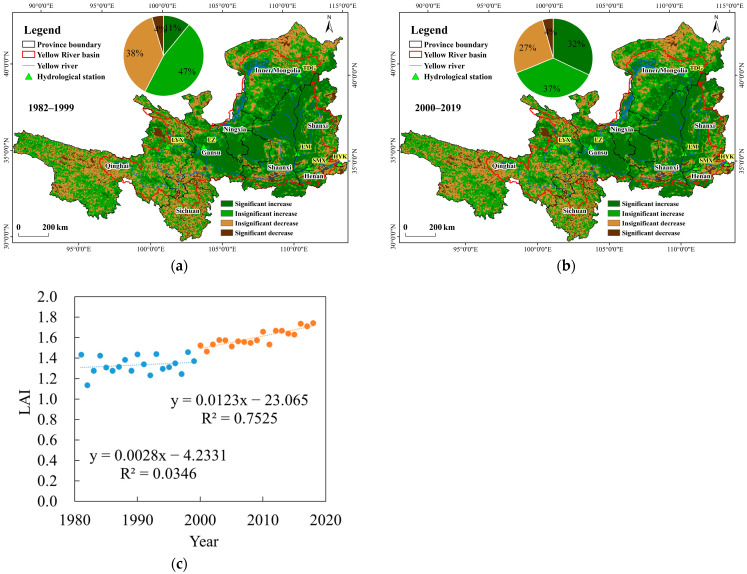
Spatiotemporal variations in LAI. (**a**) spatial distribution of LAI changes before 2000; (**b**) after 2000; (**c**) temporal changes in LAI.

Temporal trends in LAI across the Yellow River Basin are shown in Figure 9c, the LAI in the Yellow River Basin exhibited a continuous increasing trend from 1980 to 2020. The rate of increase after the year 2000 (0.0123/year) was significantly higher than that before 2000 (0.0028/year). As shown in Figure 9a,b, the spatial distribution of temporal changes in LAI across the Yellow River Basin is illustrated. During the two decades prior to the implementation of the Grain for Green Program (1980–2000), approximately 57% of the basin exhibited an increasing trend in LAI, with 11% of the area showing a statistically significant increase (*p* < 0.05). These significantly increasing areas were primarily distributed in northern Gansu and the border region of Inner Mongolia, Shanxi, and Shaanxi. Meanwhile, small-scale areas of significant decrease were observed in central Shanxi and Shaanxi during the same period. Following the implementation of the program (2000–2018), the area with an increasing LAI trend increased to 69% of the basin, and the proportion of significantly increasing areas rose to 32%. These regions were highly concentrated in the middle reaches of the Yellow River.

### 3.3. Analysis of Drivers of LAI Temporal Changes

This study employed GTWR to investigate the impacts of climatic, social, economic, and policy factors on temporal LAI changes and the spatial heterogeneity of these driving effects. To avoid the problem of multicollinearity among the initially selected factors, the 13 initial factors were subjected to OLS classical linear regression. Based on the regression results, PD, PI, PC, and PG with a variance inflation factor (VIF) greater than 7.5 were excluded. The remaining 9 factors were then analyzed in relation to LAI through the GTWR model.

#### 3.3.1. Analysis of Drivers of LAI Temporal Changes

**Figure 10:**
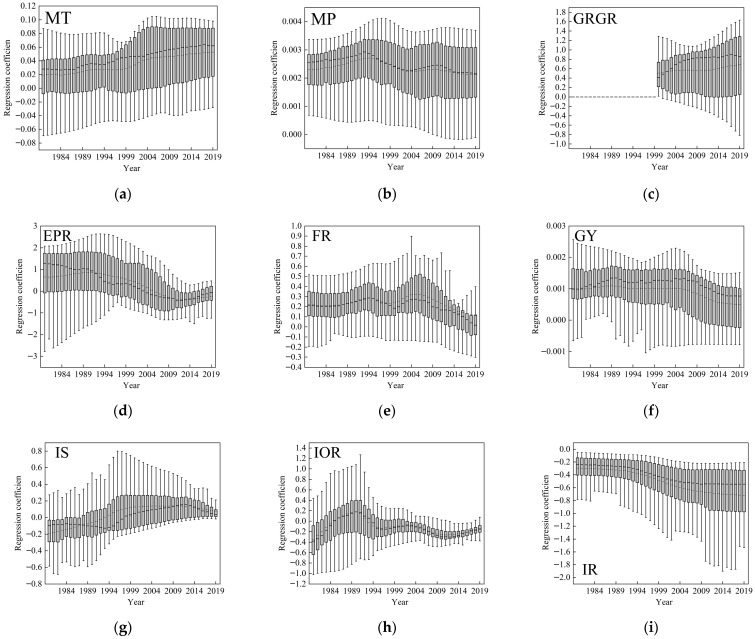
Drivers of LAI change over time. (**a**) temporal changes in MT regression coefficients; (**b**) in MP; (**c**) in GRGR; (**d**) in EPR; (**e**) in FR; (**f**) in GY; (**g**) in IS; (**h**) in IOR; (**i**) in IR.

The regression coefficients of various factors on LAI are shown in Figure 10. The regression coefficients for MT are all above zero, indicating that temperature consistently exerts a positive promoting effect on LAI. This promoting effect shows a gradually increasing trend over the study period. The regression coefficients for MP are also above zero, indicating that precipitation has a positive promoting effect on LAI in the Yellow River Basin. From 1980 to 1995, this promoting effect gradually increased, while from 1995 to 2019, it gradually weakened. EPR, FR, GY all have positive promoting effects on LAI, but these effects gradually weakened over the study period. The regression coefficients for IS were mostly below zero before 2000, indicating a negative inhibitory effect on LAI. After 2000, some of the box plots shifted above zero, suggesting that IS upgrading in certain regions has begun to positively promote LAI. The regression coefficients for IOR fluctuated below zero throughout the study period, indicating a consistently negative inhibitory effect on LAI. Similarly, the box plots for IR were mostly below zero, reflecting a consistently negative effect on LAI, with this inhibitory effect gradually strengthening over the study period. The GRGR has consistently shown a positive promoting effect on LAI, and this effect has continued to increase steadily after 2000. The mean regression coefficient rose rapidly from approximately 0.4 in 2000 to around 0.8 in 2010, after which it remained relatively stable.

#### 3.3.2. Spatial Distribution of Drivers of Temporal Changes

**Figure 11:**
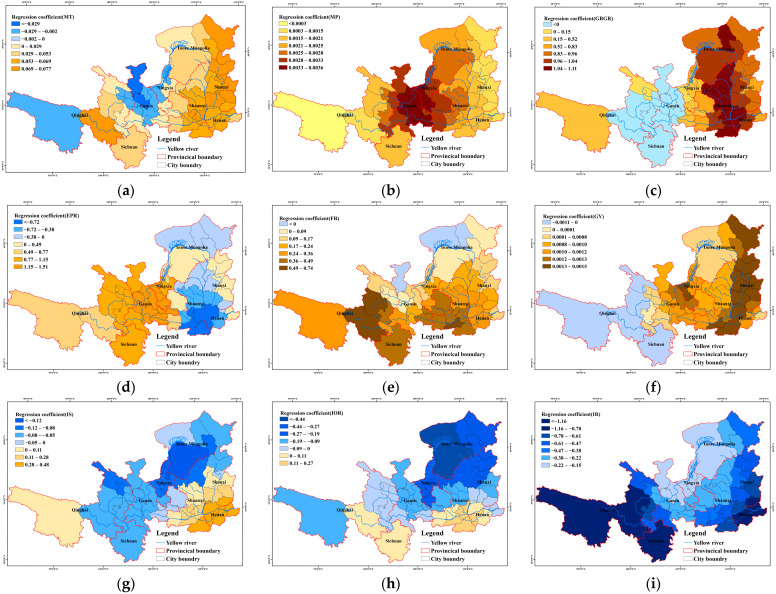
Spatial distribution of regression coefficients of driving factors for LAI changes over time. (**a**) spatial distribution in MT regression coefficients; (**b**) in MP; (**c**) in GRGR; (**d**) in EPR; (**e**) in FR; (**f**) in GY; (**g**) in IS; (**h**) in IOR; (**i**) in IR.

The spatial distribution characteristics of the regression coefficients for each factor are shown in Figure 11. The impact of MT on LAI in the Yellow River Basin is predominantly positive, with high regression coefficients mainly distributed in the eastern region. However, in northern Gansu and western Ningxia, temperature exerts a negative inhibitory effect on LAI in some areas. MP shows a consistently positive promoting effect on LAI across the entire basin, with regression coefficients exhibiting a trend of first increasing and then decreasing from east to west. The EPR on LAI displays certain spatial heterogeneity: it shows a negative effect in the eastern region and a positive effect in the western region. The FR primarily has a positive driving effect on LAI in the Yellow River Basin, with regression coefficients showing a spatial distribution pattern of higher values in the south and lower values in the north. The impact of GY on LAI is positive in the east and negative in the west, with areas of high regression coefficients mainly located in the easternmost marginal regions. The impact of IS on LAI also exhibits spatial heterogeneity, showing a positive promoting effect in the southeast and a negative effect in the northwest. The IOR mainly exerts a negative effect on LAI, with stronger inhibitory effects observed in the northern region. The IR consistently shows a negative inhibitory effect on LAI across the entire basin, with the strength of this negative effect first decreasing and then increasing from east to west.

**Figure 12:**
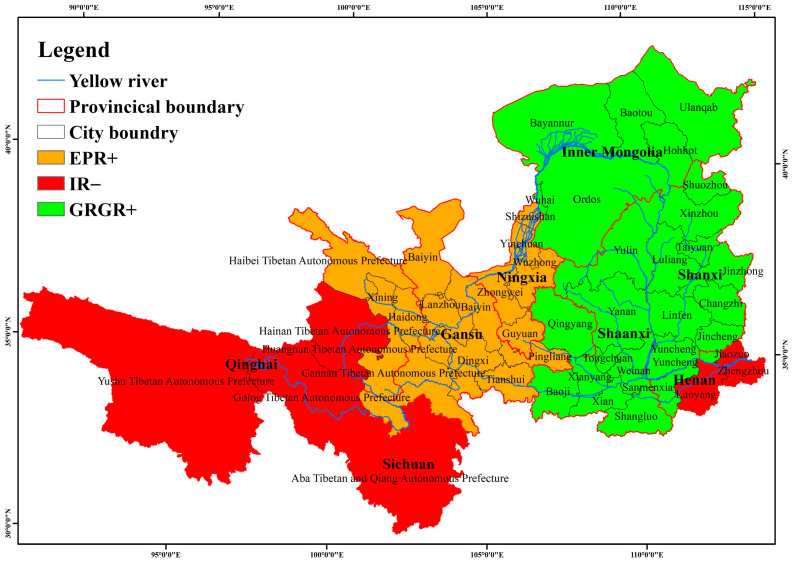
The spatial distribution of the dominant factors of LAI, where the suffix “+” indicates a positive impact on LAI, while “−” indicates a negative impact.

The dominant factors of LAI in the Yellow River Basin are illustrated in Figure 12. The primary controlling factors exhibit significant regional variations: In the eastern region, the GRGR is the main driver of LAI increase. In the central region, the dominant factor influencing LAI change is the positive effect of the EPR. In the western region, the IR has the greatest impact on LAI change, primarily showing a negative inhibitory effect on LAI.

### 3.4. Analysis of Drivers of LAI Spatial Distribution

This study utilized the Geodetector model to analyze the driving factors behind the spatial distribution of LAI in the Yellow River Basin. Factor detector was applied to calculate the q-values and significance levels of various influencing factors for the years 1980, 1990, 2000, 2010, and 2019 (Table 2). The results indicate that among natural factors, MT, MP, MD, and MS all exerted significant influences on the spatial distribution of LAI. MT had the strongest effect, followed by MS and MT, while MD showed the weakest impact. In contrast, social and economic factors generally did not exhibit statistically significant effects on LAI spatial patterns. After 2000, the GRGR significantly influenced the spatial distribution of LAI.

**Figure 13:**
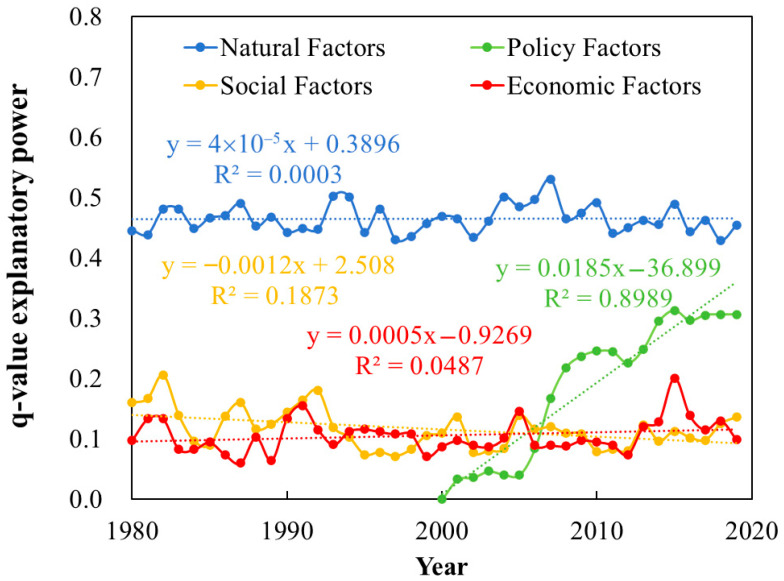
Temporal changes in the explanatory power of driving factors for LAI spatial distribution.

By calculating the average of the q-values of natural, social, economic, and policy factors, the comprehensive influence of each dimension factor on the spatial distribution of LAI was obtained, as shown in Figure 13. Natural factors exhibited the highest explanatory power regarding LAI distribution, with values consistently ranging between 0.4 and 0.5, remaining largely stable throughout the study period. Social factors showed a declining trend, with q-values decreasing from approximately 0.15 in 1980 to around 0.1 in 2019. In contrast, economic factors demonstrated a slight increasing trend, maintaining a level around 0.1 over the study duration. After 2000, the influence of the GRGR on the spatial distribution of LAI increased rapidly, with its q-value rising at a rate of approximately 0.0185 per year. Around 2006, the q-value of the GRGR surpassed that of socioeconomic factors, making it the second most important factor influencing the spatial distribution of LAI. After 2015, its q-value remained relatively stable, eventually reaching approximately 0.3.

## 4. Discussion

### 4.1. Drivers of LAI Temporal Changes

Our findings indicate that natural factors, social factors, and policy factors in the Yellow River Basin have all exerted positive effects on LAI, while economic factors have primarily shown inhibitory effects. Among these, the EPR among social factors, the IR among economic factors, and the GRGR are the most significant factors influencing LAI in the Yellow River Basin. In contrast, natural factors have had a relatively weaker impact on the spatiotemporal changes in LAI. Previous studies have mainly employed residual-based methods to separate the effects of climate and human activities on vegetation [9,22]. Although these studies did not systematically analyze different aspects of human activities, their results still suggest that human activities have a greater impact on vegetation variation in the Yellow River Basin than natural factors. This study delves deeper into specific factors across various dimensions. First, an increase in EPR reflects societal development promoting livelihood diversification, leading to a large-scale shift of rural labor from agriculture to non-agricultural sectors. This transformation led to the abandonment of farmland and the subsequent recovery of natural vegetation, thereby increasing the LAI [6,46]. Secondly, the increase in FR implies a greater government investment, with more funds allocated to environmental protection, which is conducive to enhancing the greenness of vegetation, such as through the construction of urban green spaces and ecological parks, which effectively enhance vegetation coverage [47], while, higher GY means that food demand can be met without expanding farmland through deforestation or other means, thereby promoting the greening of vegetation. The Grain for Green Program (initiated in 1999) is one of China’s most prominent ecological restoration projects. This central government-led policy explicitly prohibits agricultural cultivation on steep slopes to promote afforestation, as these areas are considered more suitable for natural trees and herbaceous plants than crops [48]. After the policy’s implementation, large areas of farmland and idle land were rapidly transformed into forests and grasslands, significantly improving regional vegetation greenness [21,22,49]. This study shows that the regression coefficient of the GRGR exhibits a gradually increasing trend. This temporal pattern not only reflects the strengthening of policy implementation over time, but also captures the inherent time-lag effects in ecological restoration. Li et al. [50] similarly found in their study on the Loess Plateau that significant ecological effects emerged 5 to 8 years after afforestation. Ding et al. [51] further demonstrated that, from a cost–benefit perspective, the maximum marginal contribution of NDVI is achieved four years after planting. Although the IS and IOR among economic factors in this study showed negative effects, their inhibitory impact gradually weakened over the research period, displaying a trend of shifting from negative to positive. In contrast, the IR consistently exhibited negative effects. Previous research has shown that economic development can negatively affect vegetation restoration. For instance, Shen et al. [52] observed a significant negative impact of economic development on vegetation in Jiangsu Province. Similarly, Wang et al. [53] reported a significant negative correlation between economic density and vegetation coverage in counties within the Yellow River Basin, with every unit increase in economic density corresponding to a 1.108% decrease in vegetation coverage. The results of this study reveal that the IR is the economic factor with the strongest inhibitory effect on LAI. This may be attributed to the relatively underdeveloped economy in the western Yellow River Basin, where non-farm employment opportunities are limited and dependence on subsistence agriculture is high [54]. A higher IR indicates rural poverty, and under the pressure of poverty, rural households tend to intensify agricultural activities, leading to vegetation degradation [55]. Our research also found that the dominant factors for LAI exhibit spatial heterogeneity. In the eastern region, the positive promoting effect of the GRGR is the most significant factor influencing the LAI increase. In the central region, the EPR is the leading factor for LAI increase. However, in the western region, the negative effect of the IR is the main factor influencing LAI changes. This reflects the complexity and heterogeneity of the socio-ecological system [4]. In conclusion, LAI temporal changes in the Yellow River Basin were predominantly driven by socioeconomic and policy factors. While regional variations in driving mechanisms exist, the results unequivocally highlight the dominant role of human activities in shaping vegetation dynamics across the basin.

### 4.2. Drivers of LAI Spatial Distribution

In terms of the spatial distribution of LAI, natural factors are the most important driving force, with a regression coefficient of around 0.5. The GRGR follows, with the regression coefficient eventually stabilizing at around 0.3. Firstly, natural factors shape the spatial pattern of vegetation by regulating regional hydrothermal conditions [56], a pattern consistent with most global ecosystems [12]. Rising temperatures significantly promote vegetation greening by enhancing photosynthesis and extending the growing season [4], while in arid and semi-arid regions, precipitation acts as the primary limiting factor for vegetation growth [57,58]. The findings of this study support this conclusion. Policy factors play a secondary role in influencing LAI spatial distribution. The Grain for Green Program has significantly altered LAI distribution through human-induced land use changes [52]. In contrast, the influence of socioeconomic factors on the spatial distribution of LAI is relatively weak. These results further indicate that LAI spatial distribution is more constrained by natural background conditions such as hydrothermal availability, and policy interventions cannot override these natural limitations. Numerous previous studies have documented cases of vegetation degradation due to afforestation practices that ignored natural conditions [59,60], For example, McVicar et al. [61] observed stunted trees in afforestation areas with annual precipitation below 400 mm; Wang et al. [62] found severe vegetation coverage degradation in forested areas converted from drylands; Qi et al. [63] reported a mere 10% forest survival rate after 40 years in the Three-North Shelterbelt Program, attributing the poor restoration outcomes to afforestation in sandy areas. Therefore, vegetation restoration should comprehensively consider natural factors such as climate, soil, and topography, and improve outcomes through rational optimization of vegetation types and spatial allocation. Luan et al. [64] demonstrated in the Yellow River Basin that artificial vegetation restoration is feasible in regions with precipitation exceeding 300 mm, whereas natural restoration is more suitable in areas with precipitation below 300 mm. He et al. [65] in their study on karst regions, found that Karst Trough Valleys and Karst Peak-Cluster Depressions, due to their superior natural conditions, are more suitable for rapid afforestation projects, whereas Karst Fault Basins and Karst Plateaus are better suited for farmland abandonment strategies. Therefore, the implementation of ecological projects requires careful consideration of local climatic, soil, and hydrological conditions [11,66], and it is crucial to develop region-specific strategies tailored to local circumstances [4].

### 4.3. Limitations and Prospects

First, this study employs the GTWR model to capture the spatiotemporal non-stationarity of LAI driving forces, with its core advantage lying in the ability to explicitly reveal the spatiotemporal heterogeneity of influence relationships. However, the GTWR model inherently assumes a linear relationship between the independent and dependent variables [44], whereas ecosystem responses to climate change and human activities often exhibit complex nonlinear characteristics. To address this limitation, future research could further introduce nonlinear modeling approaches—either for comparative analysis or integration with GTWR—such as dynamic Bayesian networks [67], and random forest [68], machine learning algorithms. This would enable a more comprehensive characterization of the driving mechanisms underlying vegetation dynamics and improve the accuracy and robustness of attribution identification.

Second, this study identifies the influencing factors of the LAI at the urban scale, which may have certain limitations. The study area encompasses multiple vegetation types, including grassland, forest, and farmland. The response mechanisms of LAI to environmental factors may differ significantly across vegetation types [69]. Modeling multiple vegetation types together may obscure the divergence in their response pathways. Therefore, future research should conduct comparative analyses by vegetation type to more precisely reveal the driving mechanisms of LAI changes across different ecosystem types.

Third, this study primarily quantifies the Grain for Green Project from the perspectives of spatial coverage and implementation intensity. However, high implementation intensity does not necessarily equate to favorable ecological outcomes. Therefore, future research should further introduce indicators that directly reflect the quality of policy implementation, such as the survival rate of revegetated areas and the rate of post-planting maintenance [70], so as to more precisely assess the actual ecological effectiveness of the Grain for Green Project.

## 5. Conclusions

This study combines LAI and climate remote sensing data with socioeconomic statistics, and uses the Geographically and Temporally Weighted Regression (GTWR) model and geographic detector to identify the key drivers of LAI changes and their spatial differentiation characteristics. The results indicate that LAI showed a continuous increase during the period from 1980 to 2019, with a significantly higher growth rate after 2000 (0.0123/year) compared to the period before 2000 (0.0028/year). Spatially, prior to 2000, approximately 57% of the basin exhibited an increasing trend in LAI, with 11% showing a significant increase. After 2000, the area with increasing LAI expanded to 69% of the basin, of which 32% experienced a significant increase. Analysis of the driving mechanisms reveals that, in terms of temporal LAI dynamics, natural factors, social factors, and policy factors primarily exerted positive effects on LAI increase, whereas economic factors mainly showed inhibitory effects. The dominant factors influencing LAI changes in the Yellow River Basin exhibit distinct zonal distribution patterns. In the eastern region, the positive promotion effect of the GRGR was the most significant factor affecting LAI changes. In the central region, the EPR emerged as the dominant factor driving LAI increase. In the western region, the negative effect of the IR was the primary driver of LAI change. Regarding the spatial distribution of LAI, both natural and policy factors were statistically significant. MP and MS had the most pronounced effects, followed by MT and GRGR, while MD had the weakest influence. The influence of socioeconomic factors on the spatial distribution of LAI is relatively weak. In summary, the LAI dynamics in the Yellow River Basin are primarily driven by human activities in terms of temporal changes, while natural factors dominate the spatial patterns.

## Figures and Tables

**Table 1:** Description of socioeconomic indicators.

Dimension	Indicator (Abbreviation)	Description	Unit
Natural	Mean Temperature (MT)	Annual average temperature	℃
	Mean Precipitation (MP)	Annual average precipitation	mm
	Mean Elevation (MD)	Average elevation above sea level	m
	Mean Slope (MS)	Average slope steepness	°
Social	Population Density (PD)	Ratio of total resident population to total city area, reflecting overall social development intensity	persons/km^2^
	Employment Population Ratio (EPR)	Proportion of employed population to total population, indicating social stability	dimensionless
	Per Capita Fixed-Asset Investment (PI)	Fixed-asset investment divided by total population, reflecting infrastructure investment	CNY/person
	Fiscal Expenditure-Revenue Ratio (FR)	Ratio of fiscal expenditure to fiscal revenue, indicating governmental investment intensity	dimensionless
	Grain Yield per Unit Area (GY)	Grain output divided by sown grain area, reflecting agricultural technical level	kg/ha
Economic	Per Capita GDP (PG)	Gross domestic product divided by total population, indicating overall economic development level	CNY/person
	Per Capita Total Retail Sales	Total retail sales of consumer goods divided by total population, reflecting market activity level	CNY/person
	Industrial Structure (IS)	Ratio of tertiary industry GDP to secondary industry GDP, reflecting industrial structure upgrading	dimensionless
	Input–Output Ratio (IOR)	GDP divided by fixed-asset investment, reflecting regional economic efficiency	dimensionless
	Urban–Rural Income Ratio (IR)	Ratio of urban disposable income per capita to rural net income per capita, reflecting urban–rural economic disparity	dimensionless
Policy	Grain for Green Ratio (GRGR)	Ratio of cumulative area under the Grain for Green Program to total cropland area, reflecting policy implementation intensity	dimensionless

**Table 2:** Explanatory power (q-value) of driving factors. * *p* < 0.05, ** *p* < 0.01.

	MT	MP	MD	MS	PD	EPR	PI	FR	GY	PG	PC	IR	IS	IOR	GRGR
1980	0.30 *	0.66 **	0.25 *	0.54 **	0.23 *	0.15	0.08	0.09	0.23 *	0.08	0.11	0.09	0.1	0.087	-
1990	0.29 **	0.72 **	0.25 *	0.51 **	0.23 *	0.06	0.2	0.06	0.16	0.048	0.16	0.12	0.16	0.18	-
2000	0.32 **	0.72 **	0.26 *	0.56 **	0.07	0.115	0.034	0.048	0.28 *	0.16	0.06	0.11	0.022	0.068	-
2010	0.30 *	0.79 **	0.261 *	0.6 **	0.06	0.07	0.13	0.03	0.09	0.11	0.06	0.18	0.04	0.06	0.245 *
2019	0.33 **	0.72 **	0.16	0.57 **	0.17	0.093	0.003	0.175	0.234 *	0.16	0.027	0.049	0.02	0.23	0.31 **

## Data Availability

The data presented in this study are available upon request from the first author.
